# Additional notes on *Ipomoea* (Convolvulaceae) in Bolivia

**DOI:** 10.1007/s12225-018-9784-z

**Published:** 2018-11-22

**Authors:** John R. I. Wood, Maira Tatiana Martinez Ugarteche, Pablo Muñoz-Rodríguez, R. W. Scotland

**Affiliations:** 1 Department of Plant Sciences, University of Oxford, South Parks Road, Oxford, OX1 3RB UK; 2 Royal Botanic Gardens, Kew, Richmond, Surrey, TW9 3AB UK; 3 Q21493730 Museo de Historia Natural “Noel Kempff Mercado”, Avenida Irala, 565 Santa Cruz, Bolivia

**Keywords:** new records, new species, Paraguay, taxonomy

## Abstract

109 species of *Ipomoea* L. are recorded from Bolivia. This total includes six new country records and two species, *I. prolifera* J. R. I. Wood & Scotland and *I. inaccessa* J. R. I. Wood & Scotland, which are described as new, while *I. jujuyensis* O’Donell is excluded having been included previously in error. The little-known *I. subalata* Hassl. is described in full and compared with *I. chondrosepala* Hallier f. and other species with which it has been confused. The paper is illustrated with line drawings, photographs and distribution maps of the main species discussed.

## Introduction

More species of *Ipomoea* L. are recorded from Bolivia than from any other country in the Americas apart from Mexico and Brazil. 102 species were listed and described by Wood *et al.* (2015) and the number has now risen to 109 as a result of further field work and herbarium studies. Although there is no easy explanation, the most important factor for this large number appears to be the latitude where Bolivia lies. Both in the northern and the southern hemispheres of the American continent, the greatest diversity in *Ipomoea* is found between 15 and 25 degrees latitude with diminishing numbers recorded from equatorial regions as well as from the more temperate zones. The diversity of habitats and the presence of several distinct biomes are clearly other factors but are unlikely to be the sole reasons, as some equatorial countries, such as Colombia, have many fewer species of *Ipomoea* despite their greater size and even more varied habitats.

Since the publication of our account of *Ipomoea* in Bolivia (Wood *et al.* 2015), we have had the opportunity to carry out fieldwork in both Bolivia and Paraguay and examine herbarium collections in both these countries and elsewhere. This has resulted in many new records of recognised species and a greater understanding of the characteristics, distribution and conservation status of a number of recently described species, especially *I. appendiculata* J. R. I. Wood & Scotland, *I. juliagutierreziae* J. R. I. Wood & Scotland, *I. lactifera* J. R. I. Wood & Scotland, *I. longibarbis* J. R. I. Wood & Scotland, *I. mucronatoproducta* J. R. I. Wood & Scotland and *I. odontophylla* J. R. I. Wood & Scotland. Also, of particular interest is a new record of *I. acanthocarpa* (Choisy) Asch. & Schweinf. from Ibiato in Cercado, Beni (*Martinez* 38), only the second record from Bolivia and this 62 years after the only previous record. We have also seen specimens from other countries of species previously thought to be Bolivian endemics including *I. chiquitensis* J. R. I. Wood & Scotland in Brazil (Wood *et al.* 2017a) and *I. mucronifolia* J. R. I. Wood & Scotland in Paraguay (Wood *et al.* 2017b).

It is now clear that we cited *Ipomoea jujuyensis* O’Donell in error for *I. squamosa* Choisy and so *I. jujuyensis* should be excluded from the list of accepted species for Bolivia. Molecular sequencing of the sole Bolivian collection, *Mendoza et al.* 2622 showed clearly that it belonged to *I. squamosa* and this was confirmed by re-examination of the specimen. The absence of *I. jujuyensis* from Bolivia, however, is odd given its presence in the Andes of Argentina, Peru and Ecuador.

## Taxonomic Treatment

The following two recently discovered species from Bolivia are new to science:

**Ipomoea prolifera** *J. R. I. Wood & Scotland*, **sp. nov.** Type: Bolivia. Dept. Santa Cruz, Prov. Vallegrande, on descent to Pampa Negra, 15 March 2018, *J. R. I. Wood, M. T. Martinez & G. Aramayo* 28441 (holotype USZ; isotypes LPB, OXF).

http://www.ipni.org/urn:lsid:ipni.org:names:60477044-2

**Fig. 1:**
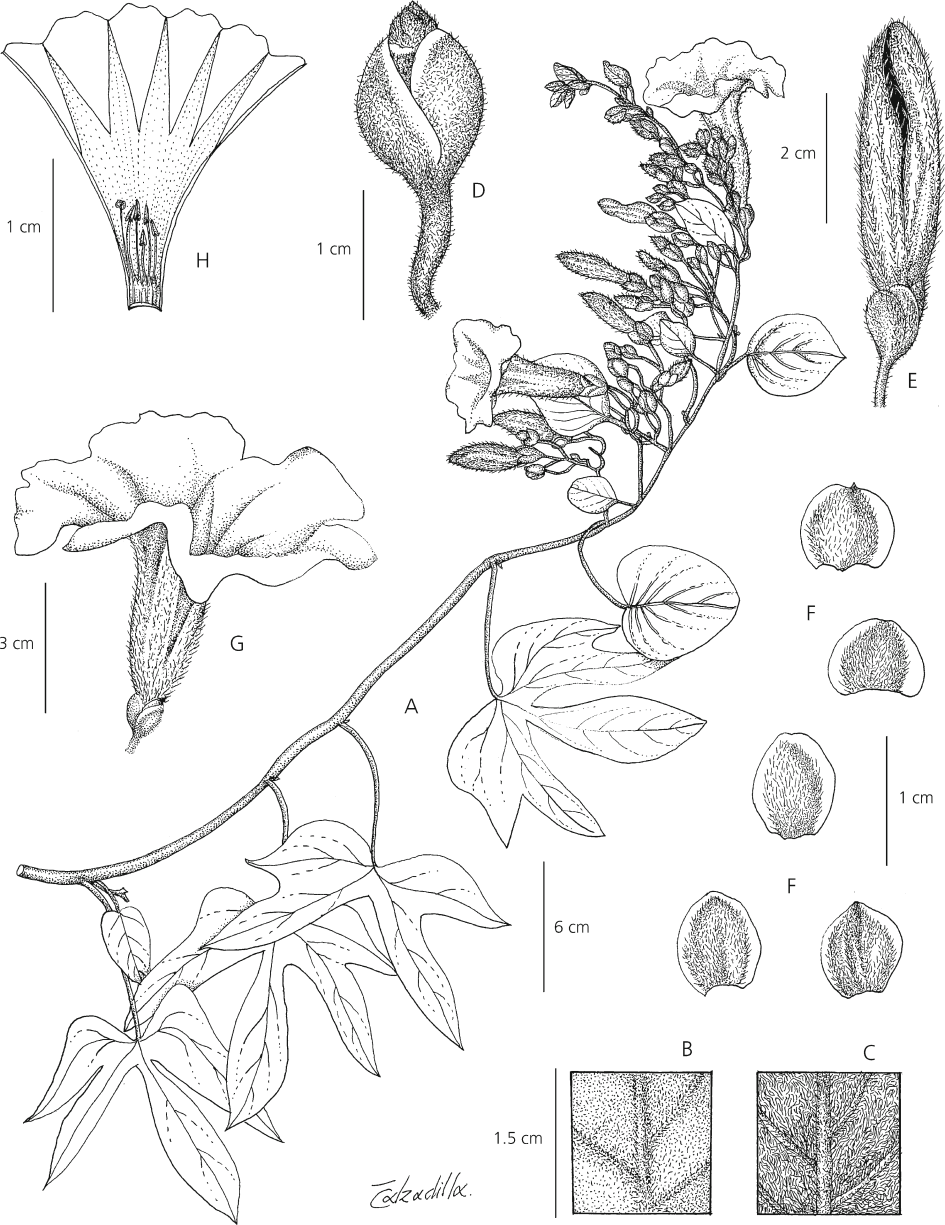
*Ipomoea prolifera.* **A** habit; **B** adaxial surface of leaf; **C** abaxial leaf surface; **D** calyx; **E** bud; **F** sepals; **G** corolla; **H** corolla opened out to show stamens and style. From *Wood et al.* 28441. drawn by eliana calzadilla.

**Fig. 2:**
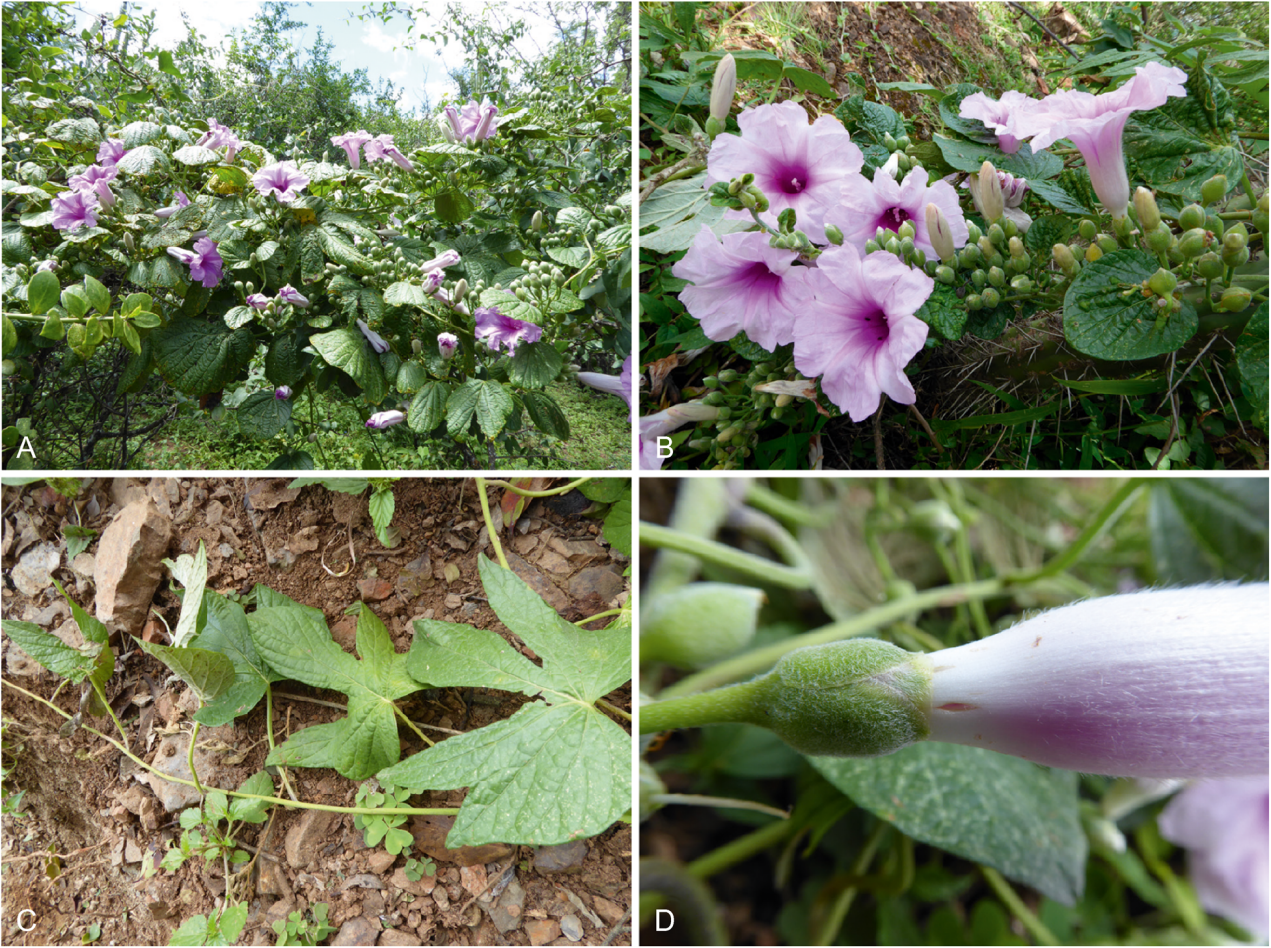
*Ipomoea prolifera.* **A** habit; **B** inflorescence with simple leaves (bracts); **C** lower palmatifid leaves; **D** detail of sepals and corolla showing indumentum. *Wood et al.* 28443. photos: maira tatiana martinez.

*Perennial herb*, clambering over shrubs or, less commonly, decumbent; stems up to c. 3 m long, pubescent with long appressed hairs. *Leaves* petiolate, dimorphic; upper leaves and bracts 2.5 – 8 × 2 – 10 cm, diminishing in size upwards, entire, broadly ovate-elliptic to suborbicular, rounded, base shallowly cordate to truncate, margins undulate; lower leaves 7 – 13 × 7 – 14 cm, 3 – 5-lobed to about halfway (rarely unequally bilobed), the lobes oblong, obtuse to acute, base shallowly cordate; both leaf forms adaxially dark green, pubescent, abaxially grey-tomentose; petioles 2.5 – 7.2 cm, pubescent. *Inflorescence* of pedunculate axillary cymes usually with 7 – 8 flowers, mainly near the branch tips, somewhat proliferating; peduncles (0.5 –) 3 – 4.5 cm, pubescent, often somewhat bent or twisted, diminishing in length towards apex; bracteoles caducous, not seen; secondary peduncles 0.5 – 2 cm; pedicels 13 – 20 mm, pubescent, often bent; sepals subequal, 8 – 9 × 5 – 6 mm, oblong-elliptic, densely pubescent, outer rounded with narrow scarious margins, inner rounded or retuse with broader scarious margins; corolla 5.5 – 6 cm long, funnel-shaped, pale pink, pubescent, limb c. 4 cm wide; stamens included, short, filaments unequal, 8 – 14 mm, glabrous except for hairs at base, anthers c. 3 mm long; style c. 20 mm long, ovary glabrous. Capsule and seeds not seen. Figs 1, 2.

**recognition**. The pubescent sepals and corolla clearly place this species in a large radiation of mainly South American species of *Ipomoea* that share these characteristics and this is confirmed by unpublished molecular data. However, the dimorphic leaves are unusual and serve to separate it immediately from species with somewhat similar palmatilobed leaves such as *I. stuckertii* O’Donell, *I. padillae* O’Donell and *I. pampeana* P. P. A. Ferreira & Miotto. *Ipomoea cardenasiana* O’Donell does present dimorphic leaves but is readily distinguished by its much larger sepals (15 – 22 mm, not 8 – 9 mm long). The subterminal inflorescence is unusual in decumbent species but is somewhat similar to that of *I. mendozae* J. R. I. Wood & Scotland but the cuneate-based, ovate to subrhomboid, entire leaves of that species are very different from the dimorphic, cordate-based leaves of *I. prolifera.* The proliferating inflorescence is also unusual. Unpublished molecular data suggest this species is closely related to *I. cuneifolia* Meisn and *I. bombycina* (Choisy) Benth. & Hook. f., but there is no obvious close morphological resemblance between these three species.

**Map 1:**
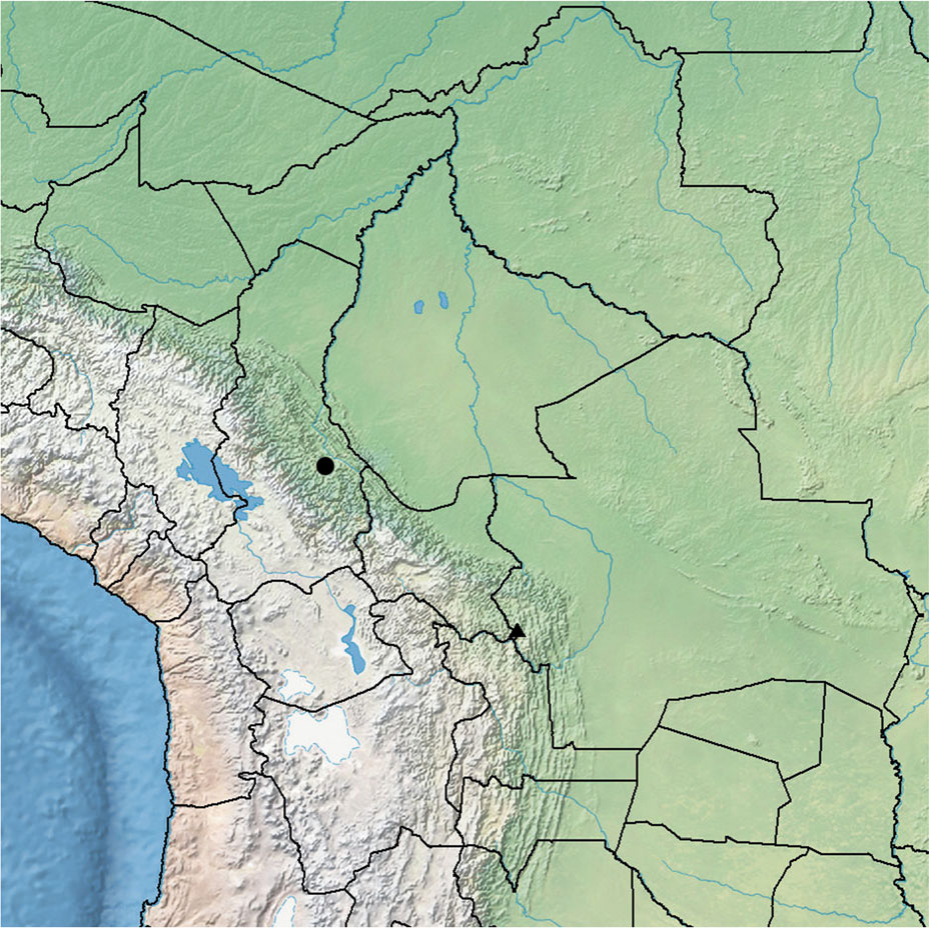
Distribution of *Ipomoea prolifera* (▲) and *I. inaccessa* (●) in Bolivia.

**distribution** **&** **habitat**. A narrow endemic restricted to seasonally very dry, deciduous, spiny bushland between 1650 and 1800 m on the descent to Pampa Negra in the Rio Mizque valley in Vallegrande Province in Bolivia. Map 1.

**specimens examined.** **bolivia**. **Santa Cruz:** Prov. Vallegrande, on descent to Pampa Negra, 18°27.746'S 64°16.322'W, 1802 m, 15 March 2018, *J. R. I. Wood et al.* 28441 (holotype USZ; isotypes LPB, OXF); ibid., 18°28.202'S 64°16.127'W, 1671 m, *J. R. I. Wood et al.* 28443 (LPB, OXF, USZ).

**conservation status**. This is a narrowly endemic species, known only from a single location, but similar dry bushland covers the hill slopes in the area and it may prove to be more common than is apparent, particularly, if as seems to be the case, it only flowers in years of good rainfall. There is no obvious threat to its habitat, so it should be classified as data deficient (DD) within IUCN guidelines until accurate population studies can be carried out.

**phenology**. Found in flower in March but probably continuing to flower into April. This plant was not seen in 2013, a relatively dry year, at the same location and in the same season, so flowering may be very dependent on adequate rainfall.

**etymology**. This species is named *Ipomoea prolifera* because of its proliferating inflorescences.

**notes**. This species is yet another endemic to the hot, dry inter-Andean valley system of the Rio Grande and Rio Mizque. Although we did not find any other endemic species in the immediate vicinity of the two collections, both *Ipomoea juliagutierreziae* and *Varronia lantanifolia* J. S. Miller & J. R. I. Wood were found nearby and other Bolivian endemic species are found at the bottom of the valley.

**Ipomoea inaccessa** *J. R. I. Wood & Scotland*, **sp. nov.** Type: Bolivia. Dept. La Paz, Prov. Caranavi, on W side of Serrania de Bellavista, above Carrasco, *J. R. I. Wood & S. G. Beck* 28539 (holotype LPB; isotypes K, OXF, USZ).

http://www.ipni.org/urn:lsid:ipni.org:names:60477045-2

**Fig. 3:**
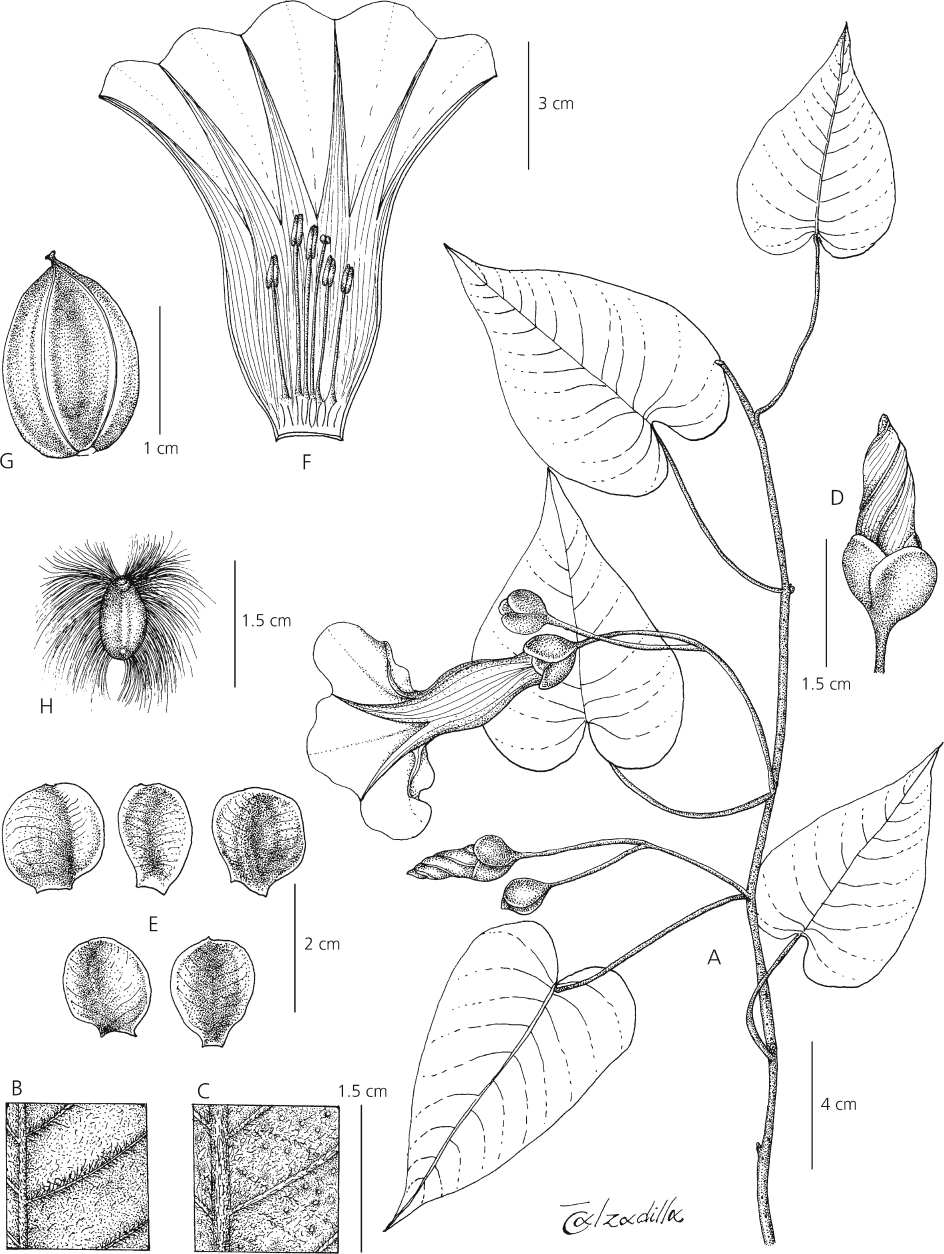
*Ipomoea inaccessa.* **A** habit; **B** adaxial surface of leaf; **C** abaxial leaf surface; **D** bud; **E** sepals; **F** corolla opened out to show stamens and style; **G** capsule; **H** seed. **A – C** from *Wood & Beck* 28539; **D – F** from *Wood & Beck* 28543; **G – H** from *Feuerer & Höhne* 4662. drawn by eliana calzadilla.

**Fig. 4:**
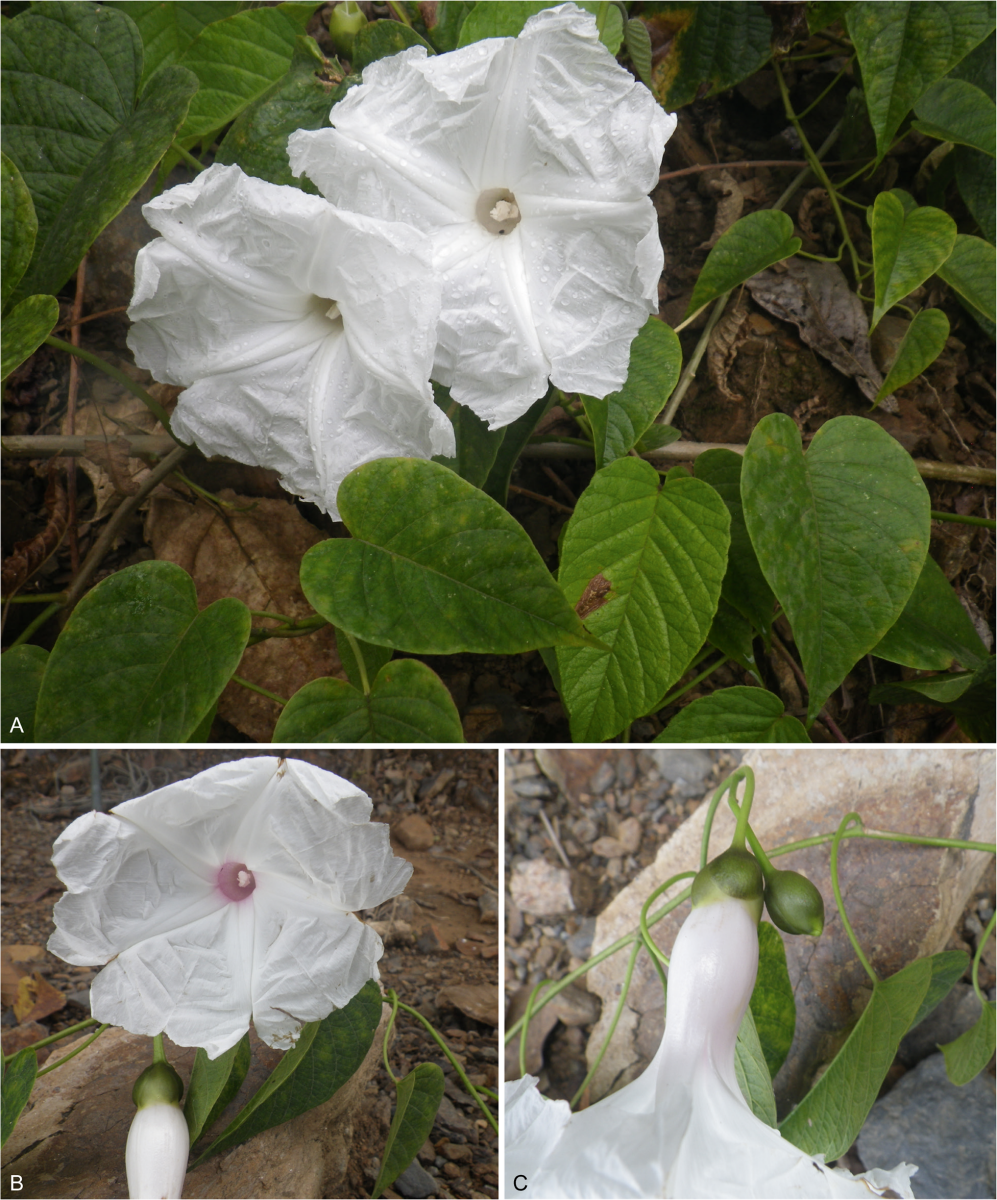
*Ipomoea inaccessa*. **A** habit of plant with pure white flowers growing on landslide; **B** corolla with pink throat; **C** detail showing sepals and corolla shape. **A**, **C** *Wood & Beck* 28543; **B** *Wood & Beck* 28539. photos: john wood.

*Liana*, 15 – 20 m high, the flowers covering the tops of trees; stems, when young green, minutely puberulous, weakly angled; when mature woody, grey, somewhat muricate, glabrescent; rootstock (juvenile) tuberous. *Leaves* petiolate, 5.5 – 14 × 3 – 8 cm, ovate, cordate, acuminate, both surfaces minutely and densely puberulent, abaxially paler with rather prominent, raised veins; petioles 2.5 – 6 cm, minutely puberulent. *Inflorescence* of (1 –) 2 – 4 (– 7)-flowered, pedunculate, axillary cymes; peduncles 2.5 – 11 cm, minutely puberulent; bracteoles at base of cyme resembling small leaves, upwards caducous, not seen; secondary peduncles 0.5 – 4 cm; pedicels 1.5 – 3.5 cm, minutely puberulent; sepals slightly unequal, somewhat convex, outer 13 – 16 × 10 mm, inner 18 – 20 × 15 – 18 mm, elliptic to subovate, rounded, rigid, glabrous, pale green with scarious margins; corolla 9 – 9.5 cm, funnel-shaped, white with pale pink throat or pure white, glabrous; limb unlobed, c. 6 cm wide; filaments unequal, 15 – 24 mm long, anthers 10 mm long; style 3 cm long; stigma biglobose. *Capsule* subglobose, 18 × 15 mm, glabrous; seeds 8 × 4 mm, pilose on the margins with hairs up to 12 mm long. Figs 3, 4.

**recognition**. A very vigorous liana reaching heights unattained by most species of *Ipomoea*. Herbarium specimens are most likely to be confused with *I. philomega* (Vell.) House but that species has smaller, deep pink corollas, 5 – 6 cm in length and, usually, glabrous leaves and distinctive reddish sepals. *I. inaccessa* has a larger corolla about 9 cm long, which is white or white with a pale pink throat. Its leaves are uniformly densely puberulent on both surfaces and the sepals are pale green. The flower colour and sepal shape suggest it is related to *I. reticulata* O’Donell and *I. saopaulista* O’Donell and this is confirmed by unpublished molecular sequence data. However, the much larger dimensions of the sepals (13 – 20 mm long, not 7 – 10 mm) and corolla (9 – 9.5 cm long, not 2.5 – 5 cm) rule out both these species.

**distribution** **&** **habitat**. Endemic to moist hill forest with frequent cloud at 1400 – 1500 m on the west side of the Serrania de Bellavista in Caranavi Province of the La Paz Department. Map 1.

**specimens examined.** **bolivia**. **La Paz**: Prov. Caranavi, on road from Caranavi to Puerto Linares, 1500 m, 5 July 1980, *T. Feuerer & N. Höhne* 4662 (LPB); Caranavi hacia Alto Beni, subiendo 9 km hacia Buenavista, 16°18'S 67°50'W, 1400 m, 5 May 1989, *S. G. Beck* 17205 (LPB); on W side of Serrania de Bellavista, above Carrasco*,* 15°42'21"S 67°29'454"W, 1420 m, *J. R. I. Wood & S. G. Beck* 28539 (holotype LPB; isotypes K, OXF, USZ); ibid.*,*15°40'49"S 67°29'524"W, 1525 m, *J. R. I. Wood & S. G. Beck* 28542 (LPB, K, OXF, USZ); ibid, *J. R. I. Wood & S. G. Beck* 28543 (LPB, K, OXF, USZ).

**conservation status**. This is a narrowly endemic species, known only from a single area of the Serrania de Bellavista. This is a large ridge, reaching about 2000 m in height, covered in moist hill forest. The habitat is vulnerable but the very steep slopes, the very moist, cloudy climate and its unsuitability for agriculture provide a degree of protection. Careful population studies are needed to assess the extent of populations of *Ipomoea inaccessa* in the area as the Serrania is extensive and mostly botanically unknown. It can only be treated as Data Deficient (DD) within IUCN guidelines until its populations are carefully studied.

**phenology**. Flowering presumably begins in March and continues into May with mature fruit present in July.

**etymology**. This species is named *Ipomoea inaccessa* because of the difficulties of collecting flowering specimens from high forest on precipitous wooded Andean slopes.

The following species are additional to those recorded for Bolivia by Wood *et al.* (2015):

**Ipomoea deminuta** *J. R. I. Wood & Scotland* (2017a: 9).

This was described as a new species based on a single collection (*E. Gutiérrez et al.* 1152) from Flor de Oro on the western side of the Rio Iténez in the Noel Kempff National Park on the Bolivian frontier with Brazil. No other collection has been seen but it might be expected to occur elsewhere in the region both in Bolivia and in Rondonia and Mato Grosso States in Brazil as the recorded habitat of flooded pampa is common in the region.

**Ipomoea longeramosa** *Choisy* (1845: 384).

This was published as a new record for Bolivia by Wood & Scotland (2017b: 6) based on *Wood et al.* 24770 (K, LPB, USZ) collected in cerrado on fine sandy soil near San Rafael in Velasco Province in the Chiquitania region of Eastern Bolivia.

**Ipomoea lindenii** *M.* *Martens & Galeotti* (1845: 264).

This species is common in Central America from Mexico southwards but is much less common in South America where it occurs sporadically along the Andes south to Peru and, now, to Bolivia. The only Bolivian collection (*N. Salinas* 3134 (LPB)) was made in Quime Province in the Department of La Paz between Comunidad Khora and Mikilpirhua at 1900 m. This collection is in fruit but shows the characteristic leaf shape, shortly pedunculate cymes and rostrate capsules typical of this species.

**Ipomoea peruviana** *O’Donell* (1948: 4).

**Fig. 5:**
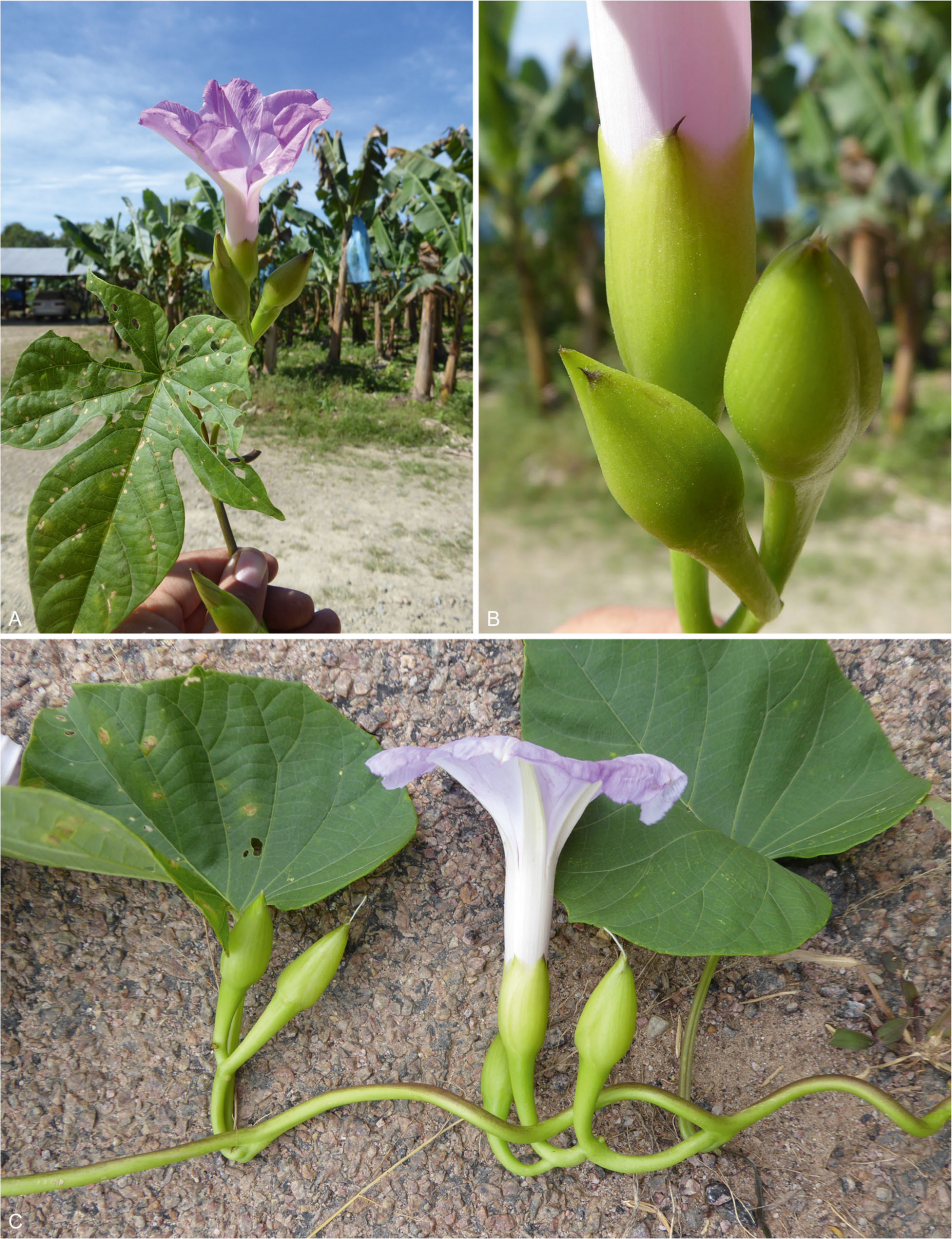
*Ipomoea peruviana.* **A** habit showing 3-lobed leaf and pale pink flower; **B** detail of sepals showing distinct mucro; **C** habit showing form with entire leaves and pale lilac flower. **A – B** *Wood et al*. 28915; **C** *Martinez & Adler* 83. photos: maira tatiana martinez.

This is known from Peru and Brazil but it was nonetheless surprising that it was found in two distinct localities in Bolivia in 2018, one Puente San Pablo, Marbán in the Beni (*Martinez & Adler* 83 [LPB, OXF, USZ]) and the other Valle de Sajta, Carrasco in Cochabamba Department (*Wood, Martinez & Ledezma* 28915 [LPB, USZ]). Both locations are in the humid lowlands in disturbed areas originally covered in rain forest. The flowers are pale lilac in colour as noted by Klug on the label of the type collection and the leaves may be entire or 3-lobed. Fig. 5.

**Ipomoea volcanensis** *O’Donell* (1953: 398).

This was regarded as an Argentinian endemic before it was collected in Bolivia in 2016. In Bolivia it is known from a single collection (*Wood, Hind & Gutiérrez* 28059 [K, LPB, OXF, USZ]) from Bosque Tucumano Boliviano at 1446 m on the ridge between Palos Blancos and Entre Rios in Tarija Department. Its occurrence in Bolivia is not unexpected but its discovery makes the absence of *Ipomoea jujuyensis* more surprising.

**Ipomoea subalata** *Hassl.* (Hassler 1911: 157).

**Fig. 6:**
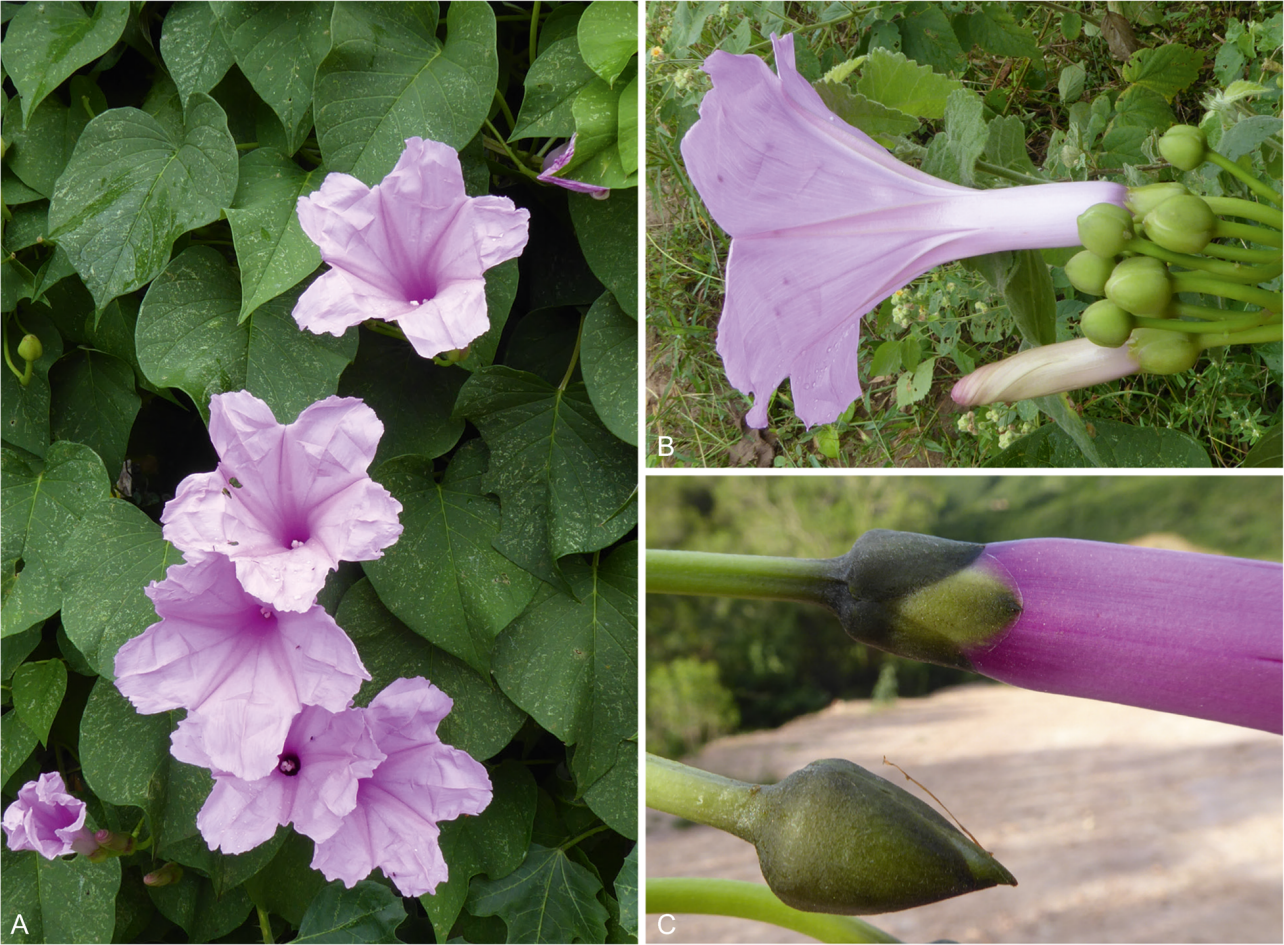
*Ipomoea subalata.* **A** habit; **B** detail showing shape of corolla and buds; **C** sepals and bud. **A – B** *Wood et al*. 28477; **C** *Wood et al.* 28431 photos: **a,** **b** roxana ledezma; **c** maira tatiana martinez.

**Fig. 7:**
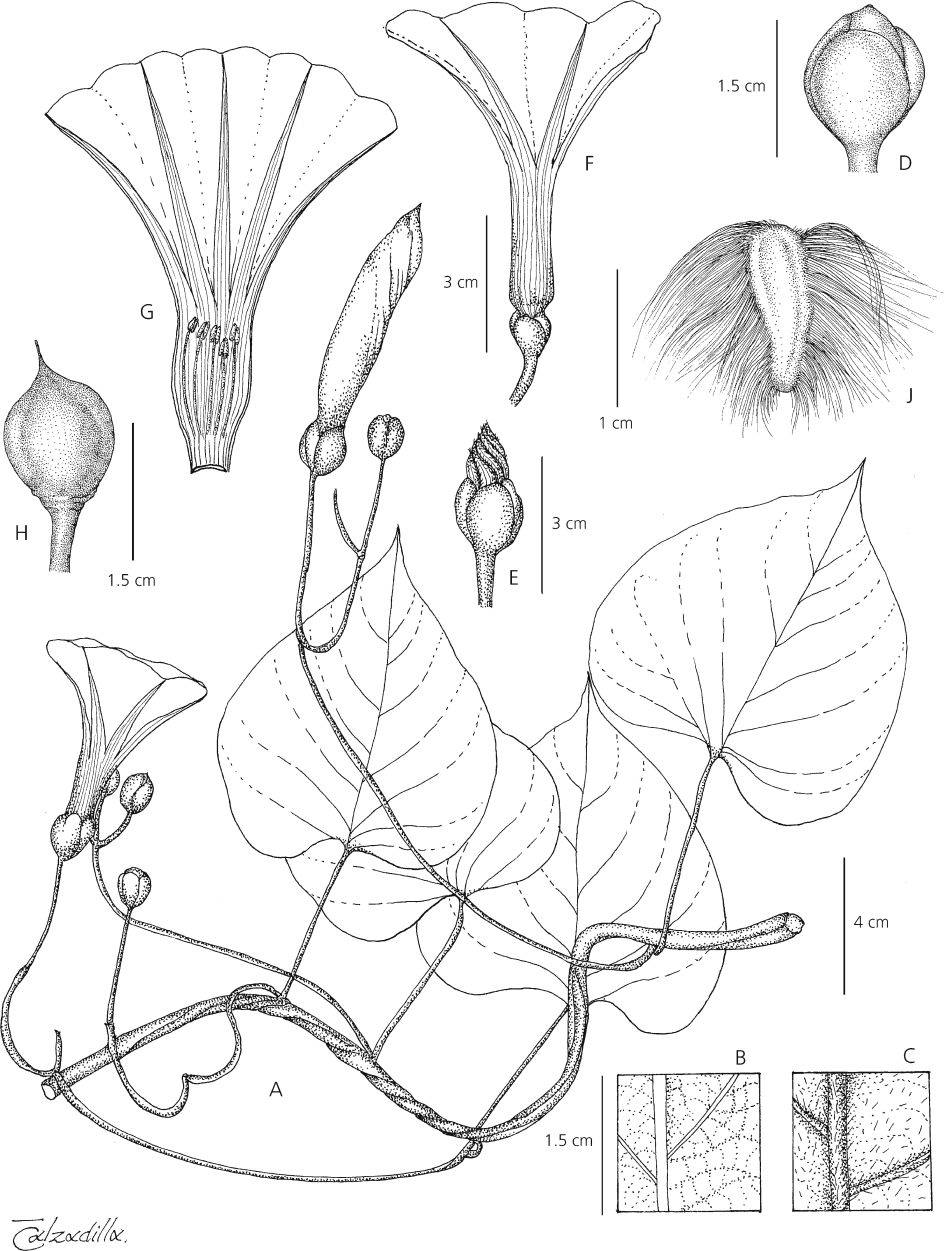
*Ipomoea subalata.* **A** habit; **B** adaxial surface of leaf; **C** abaxial leaf surface; **D** calyx; **E** bud; **F** corolla; **G** corolla opened to show stamens; **H** capsule; **J** seed. **A – G** from *Wood et al.* 27637; **H – J** from *Wood et al.* 28398. drawn by eliana calzadilla.

This is described in full below as it is a very poorly known species and is recorded for Bolivia for the first time:

Robust perennial herb reaching 6 m, stems glabrous, usually slightly winged, the wings muricate. *Leaves* petiolate, 5 – 11 × 5 – 9 cm, ovate, base broadly cordate to subtruncate, apex shortly acuminate, margin entire to undulate, often denticulate near base, adaxially glabrous, abaxially puberulent especially on the veins, sometimes glabrescent; petioles 3 – 10 cm, slightly winged below. *Inflorescence* of few-flowered, pedunculate, axillary cymes, the flower buds broadly ovoid (Fig. 6B); peduncles often erect, straight, subglabrous, 3 – 10 cm; bracteoles minute, lanceolate, caducous; secondary peduncles stout, 2 – 8 cm; pedicels 1 – 3 cm; sepals subequal, glabrous to very sparsely pubescent, margins scarious, outer sepals 10 – 12 × 7 – 9 mm, broadly ovate or elliptic, obtuse to rounded; inner sepals 11 – 13 × 8 – 9 mm, accrescent to 15 mm in fruit, elliptic or suborbicular, rounded to retuse (sometimes mucronulate), with broader scarious margins; corolla 9 – 11 cm long, funnel-shaped, pink, pubescent in bud and at tips of midpetaline bands, limb 4 – 5 cm diam., weakly lobed; stamens included, slightly unequal, very short, c. 8 – 10 mm long, style biglobose. *Capsule* 15 – 16 × 11 – 12 mm, ovoid to ellipsoid, very shortly rostrate, glabrous; seeds 6 – 11 × 3 – 4 mm, pilose on the angles. Figs 6, 7.

**Map 2:**
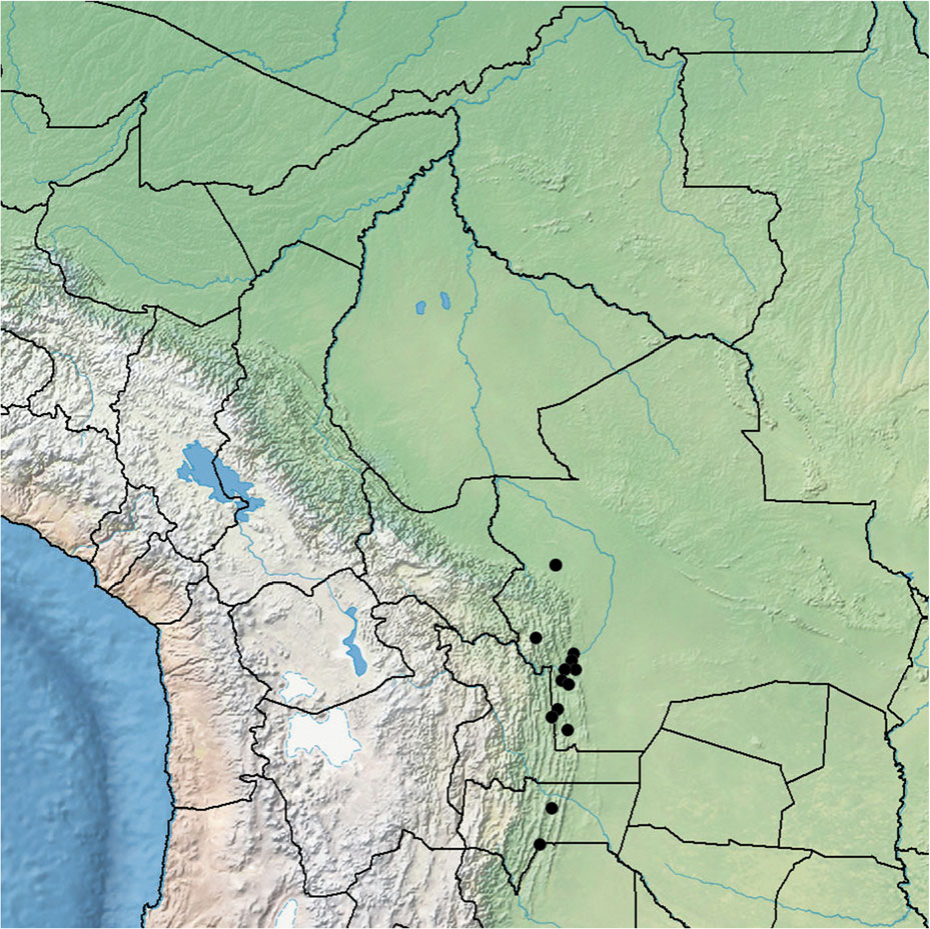
Distribution of *Ipomoea subalata* (●) in Bolivia.

**distribution** **&** **habitat**. In Bolivia, locally frequent along forest margins in xerophytic Chaco scrub and Chaco Serrano in the Andean foothills up to 1000 m (and exceptionally to 1880 m), most commonly near the town of Camiri. Outside Bolivia, known only from the type collection from northern Paraguay. Map 2.

**specimens examined.** **bolivia**. **Chuquisaca**: Prov. Luis Calvo, Mun. Villa Vaca Guzman, pie de monte de la Serrania de Incahuasi, 19°52'47"S 63°44'16"W, 1020 m, 17 Feb. 2006, *A. Lliully & Portal* 725 (HSB, MO, OXF). **Santa Cruz**: Prov. Cordillera, Tatarenda, *R. E. Fries* 1451 (S); Camiri, 800 m, Feb. 1951, *M. Cárdenas* 4734 (LIL); Abapó, camino a Com. Morocco, 18°52'01"S 63°22'52"W, 487 m, 26 April 2012, *J. R. I. Wood & F. Mamani* 27477 (K, LPB, UZ); Abapó–Laguna Tatarenda, 19°03'13"S 63°28'01"W, 786 m, 23 March 2013, *J. R. I. Wood et al.* 27590 (K, LPB, USZ); entre Ipatí y Lagunillas, 19°47'22S 63°39'38"W, 1070 m, 25 March 2013, *J. R. I. Wood et al.* 27637 (K, LPB, OXF, USZ); between Ipata and Tatarenda, 19°18'45"S 63°28'54"W, 917 m, 10 March 2018, *J. R. I. Wood et al.* 28398 (LPB, USZ); between Abapó and Laguna Tatarenda, 19°03'15"S 63°22'51"W, 769 m, 10 March 2018, *J. R. I. Wood et al.* 28399 (LPB, USZ); 5 km S of Abapó on toad to Camiri, 18°57'18"S 63°23'42"W, 492 m, 26 March 2018, *J. R. I. Wood et al.* 28477 (LPB, USZ); 10 km S of Camiri on road to Boyuibe, 20°06'14"S 63°29'13"W, 750 m, 27 March 2018, *J. R. I. Wood et al.* 28398 (LPB, USZ); 5 – 10 km from Ipata along road to Vado Yeso, 19°16' 52"S 63°32'07", 861 m, 28 March 2018, *J. R. I. Wood et al.* 28515 (LPB, USZ). Prov. Ichilo, Buenavista, c. 2 km along road to Yapacani, 17°21'11"S 63°40'24"W, 398 m, 4 April 2013, *J. R. I. Wood & B. Williams* 27735 (K, LPB, USZ). Prov. Vallegrande, c. 20 km E of Vallegrande along road to Postrervalle, 18°33'29"S 63°59'09"W, 1880 m, *J. R. I. Wood et al.* 28431 (LPB, USZ). **Tarija**: Prov. Gran Chaco, 2 km from Palos Blancos towards Villamontes, *M. Mendoza et al.* 2662 (K, USZ); Yacunda, carretera hacia Campo Largo, 22°01'16"S 63°57'27"W, 650 m, 6 March 2006, *F. Zenteno et al.* 4454 (CTES, LPB).

**conservation status**. Using Geocat (https//www.kew.org/science/projects/geocat) the extent of occupancy of this species within Bolivia is 19,744 km^2^, so it might be treated as Vulnerable (VU) according to IUCN guidelines, but based on its area of occupancy of 56,000 km^2^, it would be categorised as Endangered (EN). The categorisation as Vulnerable (VU) would seem to be more appropriate as the species thrives in disturbed scrub by roads and around “potreros” in areas of dry scrub, where there is little likelihood of significant habitat change, even though its area of occupancy is limited. Globally, this species is difficult to categorise, as the single collection from Paraguay is over a hundred years old. As no search has been made for this species in the area of the type locality, any judgement of its global status is likely to be premature. It might be extinct in Paraguay or, on the contrary, locally frequent and unthreatened given its predilection for disturbed scrubby habitats.

**phenology**. In Bolivia flowering in March and April.

**notes**. This species was described from San Luis in northern Paraguay but has never been recollected there. It has been the source of much misunderstanding by us, as well as by other botanists. In Brazil it has been equated with the plant represented by *G. & L. T. Eiten* 4077A (K, NY, SP) from Maranhão which we believe is the equally poorly known *Ipomoea cearensis* O’Donell. More recently in *Flora do Brasil 2020* (under construction) the name has been applied to *Harley* 21983, which is *I. pterocaulis* J. R. I. Wood & Scotland. In Wood *et al.* (2015: 59) we equated it with *I. megapotamica* Choisy, but this was also wrong. *I. subalata* differs in its larger corolla, longer glabrous sepals and different inflorescence. In our account of *Ipomoea* in Bolivia (Wood *et al.* 2015) we mistakenly widened our concept of *I. chondrosepala* Hallier f. (Wood *et al.* 2015: 52) to include plants whose corolla was pubescent in bud. We noted that specimens of this species from Paraguay had glabrous corollas and were uneasy about this anomaly at the time, as corolla indumentum is nearly always species-specific in *Ipomoea.* Our unease increased after observing that some Bolivian collections also had glabrous corollas but it was only after fieldwork in 2017 and 2018 in Paraguay and Bolivia, that we accepted that our concept of *I. chondrosepala* Hallier f., in fact consisted of two elements, *I. chondrosepala* itself and the little-known *I. subalata*.

The five species we know have been confused with *Ipomoea subalata* can be separated by the following key:

- 1. Corolla glabrous on the exterior; leaves and stem glabrous……………………………………………….2
  Corolla pubescent on the exterior, at least in bud and towards the tips of the midpetaline bands; leaves usually pubescent, at least on the veins beneath; stem usually thinly hirsute ……………………………………………….3
- 2. Stems winged; perennial herb; inflorescence of axillary cymes; corolla usually 8 – 9 cm long……………………………………………….**I. pterocaulis**
  Stems unwinged; woody liana, the inflorescence commonly developing on leafy side shoots; corolla 5.5 – 7 cm long……………………………………………….**I. chondrosepala**
- 3. Sepals glabrous or nearly so; corolla large, 9 – 12 cm long……………………………………………….4
  Sepals tomentellous; corolla 4.5 – 6 cm long……………………………………………….**I. megapotamica**
- 4. Stems winged; outer sepals 9 – 12 mm long……………………………………………….**I. subalata**
  Stems unwinged; outer sepals 8 – 9 mm long……………………………………………….**I. cearensis**

**Ipomoea chondrospala** *Hallier f.* (Hallier 1899: 49).

**Fig. 8:**
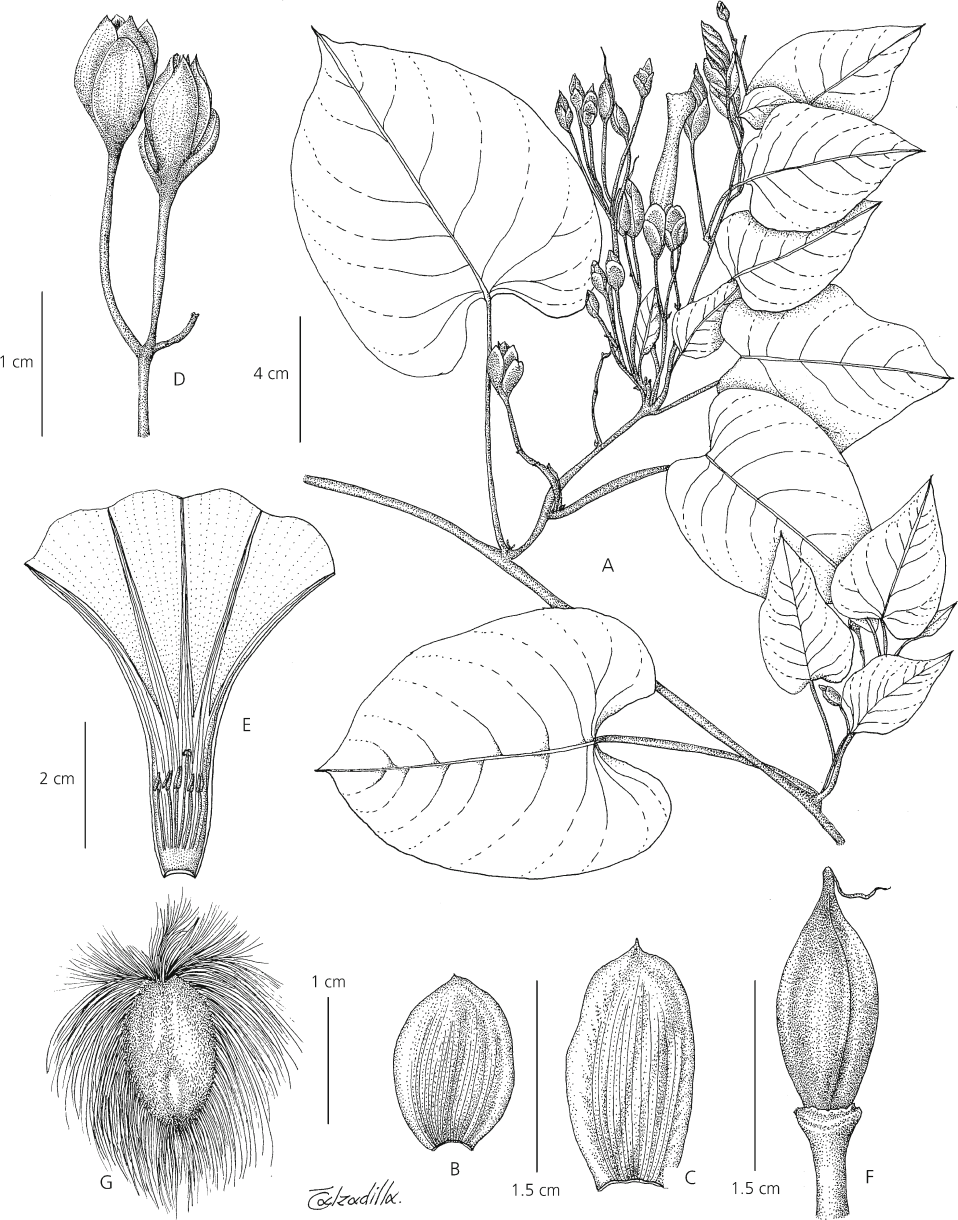
*Ipomoea chondrosepala* **A** habit; **B** outer sepal; **C** inner sepal; **D** part of inflorescence; **E** corolla showing stamens and style; **F** capsule; **G** seed. From *Wood et al.* 28286. drawn by eliana calzadilla.

Although several of the specimens cited as *Ipomoea chondrosepala* by us (Wood *et al.* 2015) are *I. subalata*, several Bolivian collections we have re-examined are definitely *I. chondrosepala* and one of these is illustrated in Fig. 8.

**Fig. 9:**
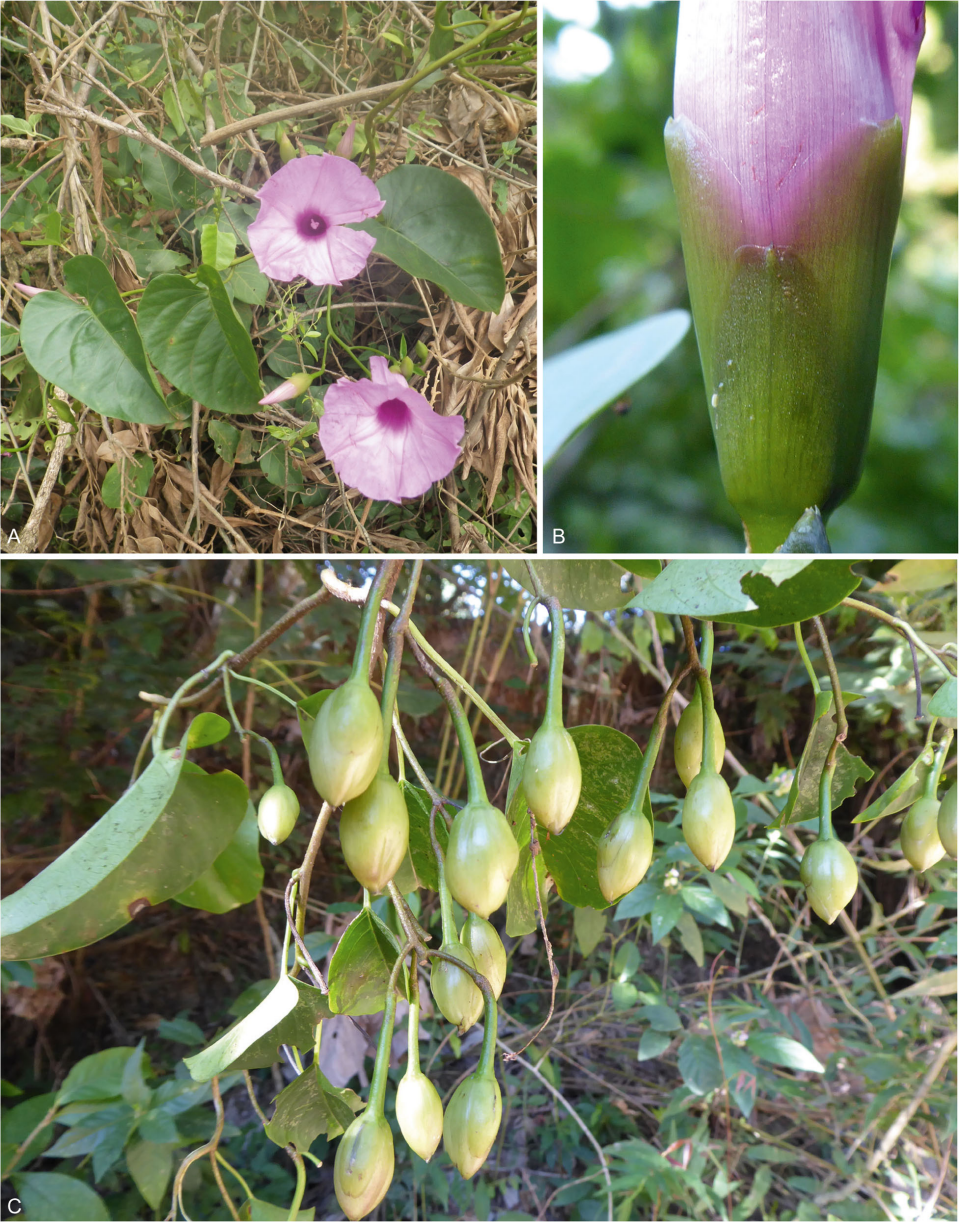
*Ipomoea chondrosepala* **A** habit; **B** detail showing greenish transparent inner sepals; **C** fruiting plant showing capsules enclosed by sepals. **A** *Wood & González* 28469; **B** *Wood et al.* 28885; **C** *Wood et al.* 28890. photos: **a** john wood; **b,** **c** maira tatiana martinez.

*Ipomoea chondrosepala* is a woody liana climbing to at least 7 m. The old stems are grey and obviously woody but the younger stems are dark green. It is completely glabrous with relatively small, somewhat shiny dark green leaves, usually 4 – 10 × 3 – 8 cm (Fig. 9A). As in several liana species, the inflorescence often develops on leafy lateral shoots, the bracteoles taking the form of small leaves. The flower buds are subcylindrical, much longer than broad, with unequal scarious, transparently green sepals, 13 – 17 × 8 – 10 mm (Fig. 9B). The corolla is only 5.5 – 7 cm long and is completely glabrous on the exterior. The sepals persist around the ripening capsule, completely enclosing it and covering it in a protective layer of water. When squeezed the water is forcefully ejected.

**Map 3:**
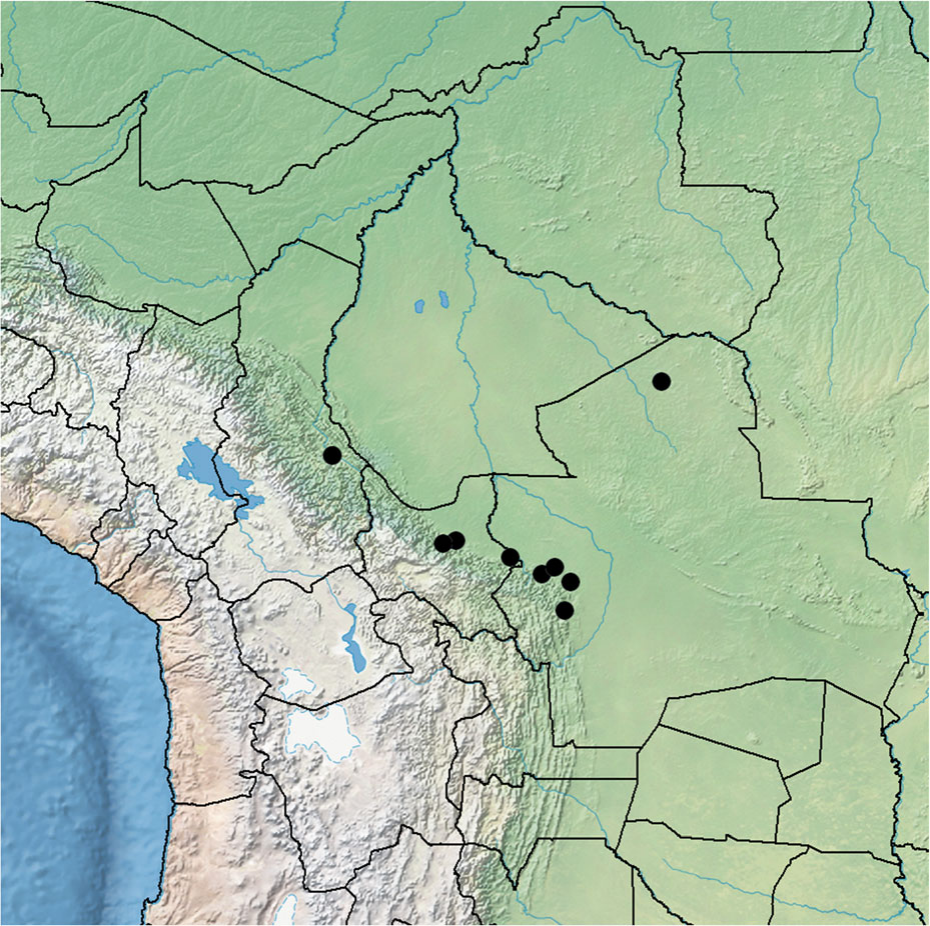
Distribution of *Ipomoea chondrosepala* (●) in Bolivia.

**distribution** **&** **habitat**. This species is recorded from Paraguay, Brazil, Peru and Ecuador besides Bolivia. In Bolivia, it is a plant of lowland subhumid or humid evergreen forest between around 200 and 800 m but prefers slightly disturbed places around tracks or clearings. It is most common in the foothills of the eastern Andean escarpment. Map 3.

**specimens examined.** **bolivia**. **Cochabamba**: Prov. Carrasco, Bulo Bulo, 17°16.404'S 64°22.957'W, 267 m, 11 July 2018, *J. R. I. Wood et al.* 28904 (LPB, USZ). Prov. Chapare, Espiritu Santo, 1891, *M. Bang* 1278 (GH, K, MO, NY, US). Prov. Tiraque, near Hotel El Puente, Villa Tunari, 16°58.955'S 65°24.464'W, 342 m, 12 July 2018, *J. R. I. Wood et al.* 28904 (LPB, USZ). **La Paz**: Prov. Sud Yungas, 2 – 3 km S of Alto Beni, Sapecho–Santa Ana, 470 m, *R. Seidel & Schulte* 2424 (K, LPB). **Santa Cruz**: Prov. Ibañez, Reserva Arubaí, 8 km de Terebinto, 17°'41'S 63°25'W, 225 m, 25 May 2006, *D. Villarroel & I. Linneo* 599 (USZ); La Angostura, 18°10'S 63°31'W, 775 m, 20 April 2002, Angostura, *M. Nee & M. Sundue* 52209 (LPB). Prov. Ichilo, P. N. Amboró, trail along Río Yapacaní towards Camp. Mataracú, 17°32'S 63°52'W, 350 m, 31 May 1998, *M. Nee & L. Bohs* 49535 (NY, USZ); near Hotel El Cafetal, Candelaria, Buenavista, 363 m, 17°27.79'S 63°41.659'W, 15 July 2017, *J. R. I. Wood et al.* 28286 (LPB, OXF); ibid., 11 July 2018, *J. R. I. Wood et al.* 28885 (LPB, USZ); ibid., near Mision Peniel, 17°28.123'S 63°41.358'W, *J. R. I. Wood et al.* 28890 (LPB, USZ). Prov. Velasco, c. 90 km S of Piso Firme on road to Santa Rosa de la Roca, 14°17'S 61°52.643'W, 25 June 2018, *J. R. I. Wood et al.* 28613 (LPB, USZ).
